# Comparison of the outcomes of stereotactic body radiotherapy versus surgical treatment for elderly (≥70) patients with early-stage non-small cell lung cancer after propensity score matching

**DOI:** 10.1186/s13014-019-1399-5

**Published:** 2019-11-07

**Authors:** Baiqiang Dong, Jin Wang, Xuan Zhu, Yuanyuan Chen, Yujin Xu, Kainan Shao, Lei Zheng, Hangjie Ying, Ming Chen, Jianping Cao

**Affiliations:** 1 School of Radiation Medicine and Protection, State Key Laboratory of Radiation Medicine and Radiation Protection, Medical College of Soochow University, Suzhou, 215123 China; 2 Institute of Cancer and Basic Medicine (ICBM), Chinese Academy of Sciences; Department of Radiation Oncology, Cancer Hospital of the University of Chinese Academy of Sciences; Department of Radiation Oncology, Zhejiang Cancer Hospital, Cancer Hospital of the University of Chinese Academy of Sciences, Hangzhou, 310011 China; 3 Department of Radiation Oncology, the First Affiliated Hospital, College of Medicine, Zhejiang University, Hangzhou, 310003 China; 4 Institute of Cancer and Basic Medicine (ICBM), Chinese Academy of Sciences; Department of Thoracic Oncology Surgery, Cancer Hospital of the University of Chinese Academy of Sciences; Department of Thoracic Oncology Surgery, Zhejiang Cancer Hospital, Cancer Hospital of the University of Chinese Academy of Sciences, Hangzhou, 310011 China

**Keywords:** Early-stage non-small cell lung cancer, Stereotactic body radiotherapy, Advanced age, Radical resection of lung cancer, Treatment outcome, Toxicity

## Abstract

**Background:**

The optimal treatment for elderly patients with early-stage non-small cell lung cancer (NSCLC) remains inconclusive. Previous studies have shown that stereotactic body radiotherapy (SBRT) provides encouraging local control though higher incidence of toxicity in elderly than younger populations. The objective of this study was to compare the outcomes of SBRT and surgical treatment in elderly patients with clinical stage I-II NSCLC.

**Methods:**

This retrospective analysis included 205 patients aged ≥70 years with clinical stage I NSCLC who underwent SBRT or surgery at Zhejiang Cancer Hospital (Hangzhou, China) from January 2012 to December 2017. A propensity score matching analysis was performed between the two groups. In addition, we compared outcomes and related toxicity in both study arms.

**Results:**

Each group included 35 patients who met the inclusion criteria. Median follow-up was 50.1 (0.8–74.4) months for surgery and 35.5 (11.5–71.4) months for SBRT. The rate of cancer-specific survival was similar between the two treatment arms (*p* = 0.958). In patients who underwent surgery, the corresponding 3- and 5-year cancer-specific survival rates were 85.3 and 81.7%, respectively. In those who received radiotherapy, these rates were 91.3 and 74.9%, respectively. Moreover, the 3- and 5-year locoregional control in patients who underwent surgery were 90.0 and 80.0%, respectively. In those who received radiotherapy, these rates were 91.1 and 84.1%, respectively. Notably, the observed differences in progression-free survival were not statistically significant (*p* = 0.934). In the surgery group, grade 1–2 complications were observed in eleven patients (31%). One patient died due to perioperative infection within 30 days following surgery. There was no grade 3–5 toxicity observed in the SBRT group.

**Conclusions:**

The outcomes of surgery and SBRT in elderly patients with early-stage NSCLC were similar.

## Background

Owing to the aging trend observed in societies and the widespread availability of low-dose computed tomographic (CT) screening, the incidence of early-stage non-small cell lung cancer (NSCLC) in elderly patients is markedly increasing. In addition, many of whom having competing comorbidities [1]. Lobectomy with lymph node evaluation has been considered the standard of care for patients with acceptable risk [2, 3]. However, surgeons are occasionally reluctant to operate in older patients due to the presence of multiple comorbidities or borderline respiratory function [4]. For this reason, the development of less radical approaches is urgently warranted.

Stereotactic body radiation therapy (SBRT) is a technique that delivers high radiation dose to a tumor target in a hypo-fractionated schedule [5]. Both the National Comprehensive Cancer Network Clinical Practice Guidelines and European Society for Medical Oncology Consensus recommend the use of SBRT as a non-surgical treatment option for stage I-II NSCLC [6]. Based on the highly promising outcome of SBRT in medically inoperable patients, several retrospective studies and prospective trials have shown that overall survival (OS) following SBRT was comparable or even better than that observed with pulmonary resection [7–12]. For elderly patients, the use of SBRT as an important alternative treatment modality has been rapidly increasing [13, 14]. However, considering the suffer from inherent imbalances in the retrospective comparison and relatively short follow-up periods of studies, there is limited data in the relevant literature comparing SBRT to surgery in this patient population.

In the present analysis, we performed a comprehensive propensity score matching analysis in order to determine the association between two potentially curative approaches for elderly patients with stage I NSCLC. This was achieved using uniform definitions of recurrence and survival from recent and ongoing clinical trials. We hypothesized that locoregional control (LRC) and cancer specific survival (CSS) in patients undergoing surgery or SBRT may be comparable.

## Methods

### Study population

In this Institutional Review Board approved retrospective study, patients aged ≥70 years, who received surgery or SBRT for T1–2 N0 M0 clinically confirmed lung cancer from January 2012 to December 2017 were eligible for inclusion. Clinically confirmed lung carcinoma was defined as a primary suspicious mass, part-solid, or ground-glass opacity nodule with spiculated or smooth margins on CT images, that persisted for ≥3 months and showed an increase in its longest axis. Patients with radiologically suspicious lymph nodes underwent endobronchial ultrasonography or mediastinoscopy. In addition, all patients underwent bone imaging and brain magnetic resonance imaging. Positron emission-computed tomography (PET/CT) was necessary for diagnosis in cases in whom biopsy was not considered medically safe or the patient refused to undergo biopsy, and recommended for all patients. Disease staging was performed using the Union for International Cancer Control TNM Classification of Malignant Tumors, 7th edition. The multidisciplinary team (i.e., surgeons, radiation oncologists, and diagnostic radiologists) examined and discussed the SBRT indications prior to the initiation of treatment. All multidisciplinary consultations were recorded in detail.

Patients with adequate pulmonary function and absence of other contraindicating medical comorbidities – according to the thoracic surgeon – were selected for surgery resection. The performance of a lobectomy, sub-lobectomy, thoracotomy, or video-assisted thoracic surgery (VATS) was discussed among the multidisciplinary team prior to the procedure. Radical lymph node dissection was performed in accordance with the current guidelines [15].

Inoperable patients – according to the thoracic surgeon – and those who refused surgical resection were selected for SBRT. The whole process of SBRT has been described previously in detail in our previous study [16, 17]. The gross tumor volume (GTV) included only the primary tumor; the internal target volume (ITV) was determined using CT with a four-dimension CT technique, and the tumor motion was assessed. The planning target volume (PTV) was defined as the ITV expanded by a 5-mm margin in each direction. The dose of SBRT was prescribed to the highest isodose line, which needed to cover 100% of the ITV and > 95% of the PTV. The treatment plans were optimized to limit the administration of high doses to regions of organs at risk. Twenty (20%) SBRT patients received more conservative fractionation schedules with a lower dose per fraction but more fractions, due to larger tumors or those adjacent to critical organs. The biological effective dose (BED) was calculated using BED_*α/β*_ = nd (1+ d/α/β), where *n* = number of fractions, d = dose per fraction, and α/β = 10 for the tumor in line with prior studies.

### Data collection

Clinical information was obtained from the electronic file database of Zhejiang Cancer Hospital (Hangzhou, China). Comorbidity scores were recorded using the Charlson Comorbidity Index (CCI). Toxicity in the SBRT group and complications in the surgery group were scored according to the Common-Terminology-Criteria-for-Adverse-Events version 4.0. To eliminate historic discrepancies in definitions of failure between surgery and SBRT, LRC was defined as the absence of any recurrence in the ipsilateral lung, the bronchial stump/suture line, and N1–N3 nodal areas. Local failure was defined as progression in the same lobe after SBRT or the bronchial stump or port site after surgery. Regional failure was defined as failure in ipsilateral hilar or mediastinal lymph nodes after either treatment. The progression free survival (PFS) indicated the length of time during and after the treatment of lung cancer that without locoregional or distant failure. The OS time was defined as the period from the date of treatment initiation to the date of death or last assessment of vital status. The CSS was defined as the interval from the date of treatment to death from recurrence or the last follow-up.

Post-treatment follow-up generally consisted of a contrast-enhanced CT scan of the thorax and abdomen performed within 2 months after treatment completion. This examination was performed on the first month, every 3 months for the first 2 years, and every 4–10 months thereafter. Primary tumor recurrence was diagnosed on the basis of histologic confirmation or enlargement of the local tumor on CT that persisted for ≥6 months. A PET-CT scan was considered when recurrence was highly suspected. When PET-CT showed a maximal standardized uptake value over 5 at half years or more after treatment, primary tumor recurrence was definitely diagnosed [18, 19]. Diagnosis of other types of recurrence was based on radiological findings of CT and/or PET-CT.

### Propensity score matching (PSM)

The propensity score was calculated using multivariable logistic regression to model a dichotomous outcome of surgery or SBRT patients. The details of patients were accessed through a database. An initial PSM analysis was performed to compare patients in the two arms based on age, gender, tumor diameter, Karnofsky Performance Status, CCI, and respiratory function, including the forced expiratory volume in 1 s (FEV1) and the ratio of FEV1 to forced vital capacity. A propensity score difference of 0.1 was used as a maximum caliper width for matching the two therapeutic groups.

### Study outcome

The main purpose of the study was to determine the LRC and CSS after treatment with SBRT or surgery in patients with early-stage NSCLC. The analyses also focused on OS, PFS, and treatment-related toxicity.

### Statistical analysis

The two-tailed *t* test was used for continuous variables, unless the data were non-normally distributed. For such cases, we used the Mann–Whitney *U* test for comparison. The *χ*^*2*^ test was used for categorical variables. The Kaplan-Meier method was used to calculate the survival rates. PSM was performed using the R MatchIt package for Windows version. A 2-tailed value of *p* < 0.05 was treated as the threshold for statistical significance.

## Results

### Patient characteristics

A total of 205 patients were selected for matching. The treatment strategy was surgical resection in 106 patients (52%) and SBRT in 99 patients (48%). Median follow-up was 51.0 (0.4–74.7) months for surgery and 30.2 (3.9–73.0) months for SBRT. In the surgical group, 90 patients (85%) underwent lobectomy, while the remaining 16 patients (15%) received sublobar resection. A total of 84 patients (79%) underwent VATS, whereas the remaining 21% underwent open thoracotomy. Mediastinal lymph node dissection or sampling was performed in all surgical patients, the number of dissected lymph nodes was 11.2 ± 5.7 (mean ± SD), with 88% of patients having 6 or more nodes dissected. All cases were margin free and R0 resections. In the radiotherapy group, patients who received SBRT exhibited significantly poorer respiratory function, higher incidence of comorbidities, and older age versus those who underwent surgery. The fractionation scheme mainly included 50 Gy in four fractions (19%), 50 Gy in five fractions (61%) and 50 Gy in ten fractions (12%). Most SBRT patients (88%) received a BED_*10*_ of at least 100 Gy. No patients in the SBRT cohort received adjuvant therapy while ten patients (9%) treated with surgery received adjuvant chemotherapy. Baseline characteristics of the patients prior to PSM are listed in Table 1.

**Table 1:** Characteristics of the entire patient cohort

Factor	SBRT (*N* = 99)	Surgery (*N* = 106)	*p*-value
Age (years)			
Median (range)	78 (70–88)	73 (70–83)	0.001
Gender			
Male	77	61	0.001
Female	22	45	
Tumor size (cm)			
Median (range)	2.2 (1.0–5.0)	2.0 (1.0–5.0)	0.621
T stage			
T1	83	86	0.740
T2	16	20	
Histology			
NSCLC-NOS	18	4	0.001
SCC	32	25	
Ade	35	77	
probable	14	0	
FEV1			
Median (range)	1.26 (0.38–2.51)	1.74 (0.78–3.04)	0.001
FEV1/FVC (%)			
Median (range)	91 (38–129)	107 (54–126)	0.001
CCI (%)			
0	45 (46)	77 (73)	0.001
1–2	48 (48)	25 (24)	
≥ 3	6 (6)	4 (2)	
KPS			
Median (range)	90 (60–100)	90 (80–100)	0.347
Tumor location			
RUL	26	29	0.323
RML	18	16	
RLL	21	20	
LUL	15	26	
LLL	19	15	
SBRT dose			
50Gy/4fx	19		N/A
50Gy/5fx	60		
50Gy/10fx	12		
60Gy/8fx	5		
70Gy/10fx	3		
Operation			
lobectomy		90	N/A
sublobar resection		16	
VATS		84	
thoracotomy		22	

*SBRT* stereotactic body radiotherapy, *NSCLC* non–small cell lung cancer, *NOS* not otherwise specified, *Ade* adenocarcinoma, *SCC* squamous cell carcinoma, *FEV1* forced expiratory volume in 1 s, *FEV1/FVC%* FEV1 to forced vital capacity ratio, *CCI* Charlson comorbidity index, *KPS* Karnofsky performance status, *fx* fractions, *RUL* right upper lobe, *RML* right middle lobe, *RLL* right lower lobe, *LUL* left upper lobe, *LLL* left lower lobe, *VATS* video-assisted thoracic surgery

PSM was performed to reduce these selection biases, and identified 35 patients from each treatment group with similar characteristics for further analysis (Table 2). The eligible patients were similar in terms of age (median age: 74 vs. 76 years, respectively), gender, tumor size (2.2 vs. 2.1 cm, respectively), and FEV1 (1.53 vs. 1.58 L, respectively). In the surgical group, 29 patients (83%) underwent lobectomy, four patients (11%) underwent segmentectomy, and two patients (6%) received wedge resection. Twenty-six (74%) patients underwent VATS, while the remaining patients (26%) received thoracotomy. Five surgical patients (14%) received adjuvant chemotherapy. Within the SBRT cohort, four patients were treated without conclusive biopsy proof of cancer when an attempt at tissue diagnosis was unsuccessful, or when a needle biopsy was not pursued based on the perceived high risk of pneumothorax.

**Table 2:** Characteristics of the propensity score-matched patients

Factor	SBRT (*N* = 35)	Surgery (*N* = 35)	*p*-value
Age (years)			
Median (range)	76 (70–83)	74 (70–83)	0.226
Gender			
Male	22	22	1.000
Female	13	13	
Tumor size (cm)			
Median (range)	2.1 (1.4–5.0)	2.2 (1.2–4.0)	0.982
T stage			
T1	28	27	0.919
T2	7	8	
Histology			
NSCLC-NOS	6	2	0.011
SCC	9	11	
Ade	16	22	
probable	4	0	
FEV1			
Median (range)	1.58 (0.55–2.40)	1.53 (0.87–2.62)	0.872
FEV1/FVC (%)			
Median (range)	101 (64–129)	103 (54–125)	0.765
CCI (%)			
0	27 (77)	24 (68)	0.503
1–2	7 (18)	9 (26)	
≥ 3	1 (3)	2 (6)	
KPS			
Median (range)	90 (70–100)	90 (80–100)	0.097
Tumor location			
RUL	9	10	0.084
RML	8	5	
RLL	7	6	
LUL	5	9	
LLL	6	5	
SBRT dose			
50Gy/4fx	8		N/A
50Gy/5fx	19		
50Gy/10fx	5		
60Gy/8fx	2		
70Gy/10fx	1		
Operation			
lobectomy		29	N/A
sublobar resection		6	
VATS		26	
thoracotomy		9	

*SBRT* stereotactic body radiotherapy, *NSCLC* non–small cell lung cancer, *NOS* not otherwise specified, *Ade* adenocarcinoma, *SCC* squamous cell carcinoma, *FEV1* forced expiratory volume in 1 s, *FEV1/FVC%* FEV1 to forced vital capacity ratio, *CCI* Charlson comorbidity index, *KPS* Karnofsky performance status, *fx* fraction, *RUL* right upper lobe, *RML* right middle lobe, *RLL* right lower lobe, *LUL* left upper lobe, *LLL* left lower lobe, *VATS* video-assisted thoracic surgery

### Survival

**Fig. 1:**
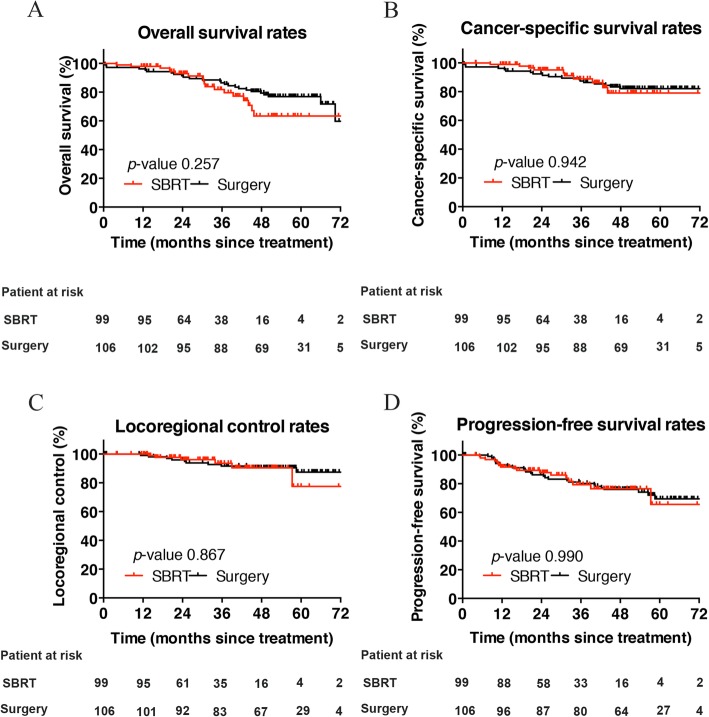
Comparison of overall survival (**a**), cancer-specific survival (**b**), locoregional control (**c**), and progression-free survival (**d**) rates of patients after surgery or SBRT prior to propensity score matching

At 3 years, the unadjusted OS, CSS, LRC, and PFS were similar between the surgery and SBRT groups (i.e., 86.5% vs. 81.8, 87.4% vs. 88.9, 93.6% vs. 91.6, and 80.2% vs. 79.4%, respectively) (Fig. 1). The PSM analysis yielded well-balanced surgical resection and SBRT cohorts. The median follow-up time was 50.1 (0.8–74.4) and 35.5 (11.5–71.4) months in the surgery and SBRT groups, respectively.

**Fig. 2:**
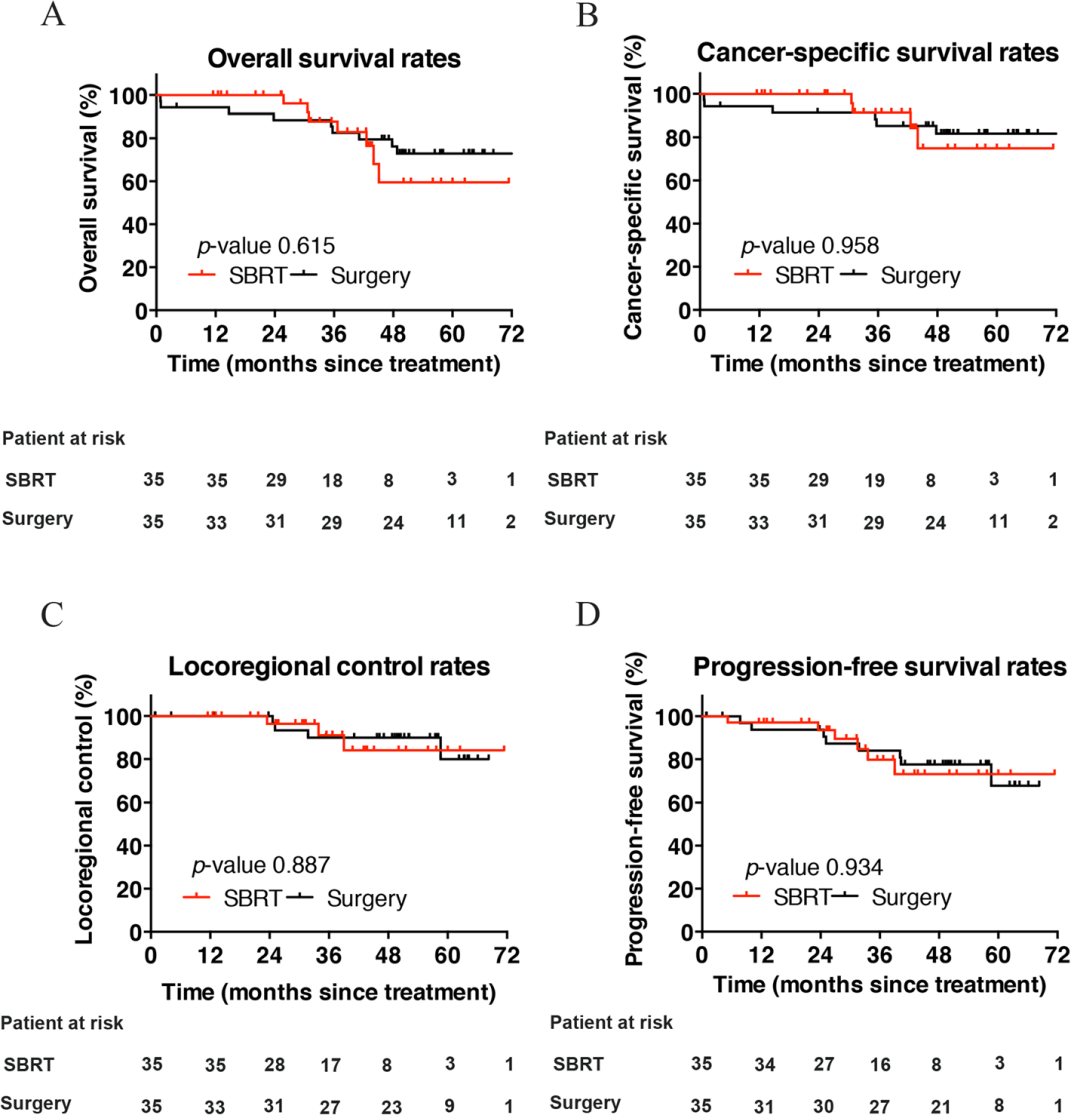
Comparison of overall survival (**a**), cancer-specific survival (**b**), locoregional control (**c**), and progression-free survival (**d**) rates of patients after surgery or SBRT following propensity score matching

After propensity matching, the 3- and 5-year OS rates in patients who underwent surgery were 82.5 and 72.9%, respectively. In the SBRT group, these rates were 87.8 and 59.5%, respectively. Notably, survival was not significantly different between the two groups (*p* = 0.615). Kaplan–Meier plots comparing patterns of survival for the entire cohort of patients are presented in Fig. 2a.

There were 16 patients (23%) who died during the follow-up period (median: 33.2 months). This was due to recurrence of lung cancer in 10 cases (14%) and other causes in six cases (9%); the latter cases included pneumonia (*n* = 2), cardiovascular disease (*n* = 2), and death of unknown cause (*n* = 2). The CSS was similar between the two treatment groups (*p* = 0.958). The corresponding 3- and 5-year CSS in the surgery were 85.3 and 81.7%, respectively. In the SBRT group, these rates were 91.3 and 74.9%, respectively (Fig. 2b).

Local failures in both groups were uncommon. In the surgery group, one patient developed a bronchial stump failure and one additional patient developed a port site failure. In the SBRT group, two patients developed primary tumor failure. Two patients in each group developed regional failure. Locoregional recurrence occurred in seven patients, four in the surgery group who received lobectomy and three in the SBRT group, with one of them received BED_*10*_ < 100 Gy. The LRC rates did not differ significantly between the groups (*p* = 0.887). Among patients who underwent surgery, the LRC rates at 3 and 5 years were 90.0 and 80.0%, respectively. In the SBRT group, these rates were 91.1 and 84.1%, respectively (Fig. 2c).

Distant metastasis was reported in 12 patients (i.e., seven in the surgery group and five in the SBRT group); the majority of cases (*n* = 8) were intrapulmonary metastases. The 3- and 5- year PFS for matched patients receiving surgery were 84.1 and 67.9%, respectively, compared to 79.8 and 73.1% for SBRT. However, the observed differences in the PFS rates were not statistically significant (*p* = 0.934) (Fig. 2d).

### Treatment toxicity

Grade 4 and 3 toxicity was reported in 2 and 4 cases, respectively, in the whole unadjusted cohort. The 30-day surgical mortality was 2/106, one additional patient died at 3 months, while no deaths were attributed to SBRT. Overall patterns and degrees of toxicity were different between the two treatment groups, and were not comparable statistically.

After PSM, none of the patients who received SBRT experienced grade 3–5 toxicity. Systemic reactions were mainly fatigue, anorexia, and dyspnea during treatment. Most of these reactions resolved after symptomatic treatment. Grade 1–2 toxicity within 6 weeks after SBRT was observed in four patients (11%). There was no occurrence of grade 3–5 radioactive pneumonitis. In the surgery group, grade 1–2 complications were observed in eleven patients (31%). Of note, one patient died due to perioperative infection within 30 days after surgery.

## Discussion

The prevalence of NSCLC in elderly patients is expected to increase further in the future. In clinical practice, treatment decisions for this population should consider the patient’s life expectancy, presence of comorbidities, estimated benefits and treatment risks, and patient preferences [20]. Guidelines established by the American Society for Radiation Oncology and the American Society of Clinical Oncology recommend a multidisciplinary approach, with shared decision-making between physicians and patients in the management of early-stage NSCLC [19, 21]. The current recommendations support the role of SBRT in patients with a high surgical risk, including those with predicted FEV1 < 50%, predicted carbon monoxide diffusing capacity < 50%, or a combination of age, impaired lung function, pulmonary hypertension, and poor left ventricular function. The median age of patients included in numerous studies describing SBRT outcomes in early-stage NSCLC was approximately 70 years [10, 22, 23]. In contrast, research focused on lung SBRT in the elderly is sparse, results obtained from randomized control trials are inconclusive. This has resulted in minimal data to guide the decisions of clinicians.

Retrospective reviews, such as the present study, provide further evidence regarding the potential role of SBRT in the treatment of older early-stage patients with high rates of long-term LRC and limited toxicity. Our aim was to compare the outcomes of surgery and SBRT in older adults with early-stage lung carcinoma, using propensity scores to control for selection bias and a coherent definition of failure in both groups. The data indicated a significant difference in the pulmonary function of patients treated with surgery and SBRT. In addition, patients with more competing comorbidities tended to be allocated to SBRT. However, unadjusted analyses did not reveal differences in 3-year OS, CSS, LRC, and PFS between the surgery and SBRT groups.

We subsequently used PSM to balance the distribution of measured prognostic factors across the study arms. Both cohorts were similar in terms of age, gender, respiratory function, and CCI. The results of this study indicated that the 3- and 5-year LRC and CSS linked to SBRT are comparable with those reported after surgery, which were consistent with the conclusions of those previously reported. A North America population-based study utilizing the SEER-Medicare database to evaluate outcomes for 10,923 patients ≥66 years of age with early-stage NSCLC from 2001 to 2007 and used PSM to control for baseline characteristics. Treatment distribution was lobectomy (59%), sublobar resection (11.7%), conventional radiation (14.8%), observation (12.6%), and SBRT (1.1%). In comparisons with surgery, patients receiving SBRT showed similar OS and CSS [24]. Another analysis of the SEER-Medicare database identified 9093 patients, aged 65 years or older, with early-stage NSCLC treated between 2003 and 2009 with lobectomy, sublobar resection or SBRT. The treatment distribution was the following: 79.4% of patients underwent lobectomy, 16.5% sublobar resection, and 4.2% SBRT. After PSM was performed, lobectomy patients performed better than sublobar resection, but had equal survival outcomes to SBRT [12]. Two analyses using uniform definitions of locoregional failure have reported better local control rates with surgery, however regional and distant control rates appear to be similar [25, 26]. Chen et al. recently conducted a meta-analysis, including only propensity score studies about surgery versus SBRT comparison. Despite favorable results in terms of OS reported in surgical subgroups, two treatment methods did not show any difference in terms of CSS [27].

The lack of a significant difference in OS may be attributed to the shorter life expectancy and higher possibility of non-cancer-specific mortality among elderly individuals. An alternative explanation may be the differences in subsequent treatments. Patients who received SBRT were in a worse condition versus those who underwent surgery at the time of disease recurrence, may often necessitating treatment with less toxic but efficacy tyrosine kinase inhibitors. The relative survival benefits of this salvage therapy might explain the OS similarity. Another possible reason may be that the sample size after PSM was insufficient, reducing the statistical significance. While many retrospective institutional studies, including those meta-analysis, have noted patients undergoing surgery to have longer OS compared to SBRT patients particularly when the operation performed is a lobectomy [25, 28–30], others have found equivalent overall survival when treatment groups were adjusted for variables that might lead to a selection bias [25, 31–33]. Palma and colleagues conducted a population-based matched-pair comparison in the Netherlands, evaluating patients ≥75 years with early-stage NSCLC. Sixty patients receiving SBRT were matched with sixty patients undergoing surgery (82% undergoing lobectomy). One and 3-year OS rates were not significantly different between both treatments [14]. Overall these studies evaluating surgery and SBRT suggest that SBRT may be a reasonable and equivalent treatment modality in comparison to surgery, though further prospective data is needed, specifically in the older adult population.

In this analysis of elderly patients, the OS in the SBRT arm is quite high and uncommon, considering that the baseline pulmonary function and capacity status of radiotherapy patients was generally better than those in the aforementioned series, which may due to the small patient numbers of subpopulation. Several reports have confirmed that poorer pulmonary function and performance status are correlated with worse OS [34, 35]. The study by Haasbeek et al. limited the analysis to patients ≥75 years, and pretreatment FEV1% with a cutoff of 50% was significantly associated with OS in the multivariate analysis [36].

Pathological confirmation was available for 100% post-PSM surgery patients and 89% post-PSM SBRT patients, which deserves further discussion. Although pathological confirmation of disease should be sought wherever possible, four patients in the SBRT group did not undergo biopsy because the procedure was not considered medically safe or due to patient refusal. In published SBRT series or in routine clinical practice, not all patients have pathologic confirmation of NSCLC prior to SBRT [37]. The clinical diagnosis of NSCLC in these cases is often based on the patient’s clinical history (e.g., tobacco use) and imaging examinations [38]. The national guidelines regarding the use of radiotherapy in the Netherlands indicate that patients without histologic confirmation undergo radiotherapy in case of: (a) the presence of a new or growing lesion on CT scans with characteristics of malignancy; (b) a high risk for the development of lung cancer based on age and smoking history; and (c) PET/CT-positive lesions [39]. The probability of benign disease in these patients is merely 4.3% [40].

In the present study, segmentectomy (11%) and wedge resection (6%) were also performed in patients at a high risk of lobectomy due to a low pulmonary function or the presence of other severe comorbidities. Recently, enthusiasm regarding the use of sublobar resection instead of lobectomy in elderly patients has increased [41]. While ongoing surgical trials attempt to identify the role of sublobar resection in stage I NSCLC [42, 43], the predominance of lobectomy in this series highlights our institutional bias towards lobar resection, reserving sublobar resection for high risk patients. In the post-PSM surgical group, the vast majority of patients (74%) underwent VATS. The introduction of minimally invasive methods for the resection of lung cancer has resulted in a significant impact on patient care and outcomes. In one study, compared with traditional thoracotomy, VATS was associated with decreased pain, shorter hospitalization, fewer perioperative complications, and fewer blood transfusions [44]. VATS lobectomy has been studied in high-risk populations of patients who may be considered reluctant for surgery, including the elderly and those with inadequate respiratory function. In these groups of patients, superior perioperative outcomes have been found with VATS lobectomy [45].

Toxicity is particularly important when considering options for the treatment of cancer with similar long-term survival. Studies investigating the use of SBRT in elderly patients have yielded mostly favorable results. In this study, we observed very limited toxicity in the two groups. Of note, one matched patient (3%) died due to perioperative infection within 30 days after surgery, no deaths were attributed to SBRT (*p* = 0.918). A larger retrospective population-based analysis identified 4235 elderly patients (≥67 years) with early-stage NSCLC treated with surgery (3852) or SBRT (383) between 2007 and 2009. After 2:1 PSM, a total of 711 surgical patients were evaluable to 367 SBRT patients. Acute complications, primarily infectious or respiratory in nature, within 1 month were more common in the surgical group (55% vs 8%), but chronic complications were similar. Mortality within 3 months was higher in surgery (6% vs 2%), but lower at 24 months (22% vs 40%). The study concluded that for patients with short life expectancies, SBRT may be preferable, while patients with a life expectancy greater than 5 years may have a survival benefit from surgery [46]. A retrospective study looked at outcomes of 24 octogenarian patients treated with radiation doses ranging from 48 to 56 Gy given in 4–5 fraction. The authors reported favorable results, with a 2-year disease free survival rate of 77%, and a 100% local control rate, no grade 3–5 treatment toxicities occurred. Despite the small sample size, this study provides data suggesting that SBRT is safe and effective, even in patients ≥80 years old [47]. For the older population with increasing age-related comorbidities, the role of SBRT as a curative modality for early-stage NSCLC may become a more attractive option, given the comparable outcomes and low rates of treatment-related morbidity and mortality versus surgery.

Currently, there are no data available from prospective trials regarding the effectiveness of SBRT in the elderly. Our study complements the existing body of early-stage NSCLC literature by providing evidence that may be relevant for patient ineligible for randomized trials. A strength of the present analytic approach is that the demographic and tumor-matching factors were comprehensive, with limited variability at baseline. The strict 0.1 maximum caliper width for propensity score difference guaranteed an accurate PSM. Another advantage is the strict unified definition of recurrence and survival. Several studies have suggested that inconsistent definitions of locoregional failure between two groups may lead to different outcomes. Furthermore, the patient population truly reflected clinical practice. The study population was not composed of selected, relatively suitable patients, which is often the case in clinical trials. Another strength of the present study is the longer follow-up versus those performed in previous studies.

The limitations of this study must be acknowledged. Although the cohorts were accurately matched, it remains a retrospective study, unidentified or unrecorded factors may have played a role in selected patients. Matched patients in the SBRT cohort had significantly more squamous cell carcinomas compared with surgery cohort in this study, which might have negatively influenced prognosis. Studies have shown that compare with adenocarcinoma, squamous cell carcinoma and low-grade differentiation are associated with worse LRC and OS [48, 49]. Another criticism of the present study is that in this more heterogeneous population, surgical patients underwent not only lobectomy (83%) but sublobar resection (17%). Inclusion of mixed extents of resection may preclude a meaningful comparison because of differences in long-term OS between lobectomy and sublobar resection for early-stage NSCLC [12]. Mixed surgical approaches (VATS vs. open thoracotomy) may also influence as a bias [50]. Moreover, five (14%) matched SBRT patients received suboptimal doses (BED_*10*_ < 100 Gy) due to large tumors or those adjacent to critical organs, and one case eventually developed into a regional failure. Data from the literature showed that poor local control and survival after SBRT is related to BED of radiation less than 100 Gy [51–53]. However, the limited number of total events in this study makes difficult to explore a correlation between dose and LRC. We should also note that the treatments for recurrence and adjuvant chemotherapy in the both groups might have contributed to improved outcomes but were not analyzed in this study. The relatively small sample size of the patient cohort is another limitation and therefore, the results have to be interpreted with caution.

## Conclusions

The management of elderly patients with early-stage NSCLC poses a unique challenge. Our analysis demonstrated that the effectiveness of SBRT in elderly patients is promising, based on the lower risk for periprocedural mortality and encouraging long-term survival versus surgery. Large randomized trials will ultimately be required to accurately compare outcomes between these therapeutic approaches.

## Data Availability

The datasets used and/or analyzed during the current study are available from the corresponding author on reasonable request.

## Abbreviations

CCI: Charlson Comorbidity Index
CSS: Cancer-specific survival
CT: Computed tomographic
FEV1: Forced expiratory volume in 1 s
FEV1/FVC%: FEV1 to forced vital capacity ratio
fx: Fraction
GTV: Gross tumor volume
ITV: Internal target volume
KPS: Karnofsky performance status
LLL: Left lower lobe
LRC: Locoregional control
LUL: Left upper lobe
NSCLC: Non-small cell lung cancer
OS: Overall survival
PET/CT: Positron emission-computed tomography
PFS: Progression-free survival
PSM: Propensity score matching
PTV: Planning target volume
RLL: Right lower lobe
RML: Right middle lobe
RUL: Right upper lobe
SBRT: Stereotactic body radiotherapy
VATS: Video-assisted thoracic surgery
